# Risk factors for quinolone-resistant *Escherichia coli* infection: a systematic review and meta-analysis

**DOI:** 10.1186/s13756-019-0675-3

**Published:** 2020-01-09

**Authors:** Dong-Mei Zhu, Qiu-Hong Li, Yan Shen, Qin Zhang

**Affiliations:** 1 Department of Hospital Infection Control, Chongqing Health Center for Women and Children, 120 Longshan Road, Chongqing, Chongqing, 400013 China; 2 Department of Clinical Laboratory, Chongqing Health Center for Women and Children, Chongqing, Chongqing, 400013 China

**Keywords:** Quinolone, Resistance, *Escherichia coli*, Infection, Risk factor, Meta-analysis

## Abstract

**Background:**

Antimicrobial resistance to quinolone is rising worldwide, especially in *Escherichia coli* causing various infections. Although many studies have been conducted to identify the risk factors for quinolone-resistant *Escherichia coli* (QREC) infection, the results are inconsistent and have not been systematically reported. The aim of the present study is to conduct a systematic review and meta-analysis to evaluate the potential risk factors for QREC infection.

**Methods:**

A systematic search was performed to collect published data in the EMBASE, PubMed, and the Cochrane Library up to April 2019. Risk factors were analyzed using the pooled odds ratio (ORs) with 95% confidence interval (CIs).

**Results:**

Twenty-seven trials involving 67,019 participants were included in the present study. The following risk factors associated with QREC infection were identified: (1) male (OR = 1.41), (2) hepatic cirrhosis (OR = 2.05), (3) diabetes mellitus (OR = 1.62), (4) cardiovascular disease (OR = 1.76), (5) neurogenic bladder (OR = 8.66), (6) renal dysfunction (OR = 2.47), (7) transplantation (OR = 2.37), (8) urinary tract infection (OR = 2.79) and urinary tract abnormality (OR = 1.85), (9) dementia (OR = 5.83), (10) heart failure (OR = 5.63), (11) neurologic disease (OR = 2.80), (12) immunosuppressive drugs (OR = 2.02), (13) urinary catheter (OR = 4.39), (14) nursing home resident (OR = 4.63), (15) prior surgery (OR = 2.54), (16) quinolones (OR = 7.67), (17) other antibiotics (OR = 2.74), (18) hospitalization (OR = 2.06) and (19) nosocomial infection acquisition (OR = 2.35).

**Conclusions:**

QREC infection was associated with nineteen risk factors including prior quinolones use, hospitalization, and several comorbidities. Reducing exposure to these risk factors and modification of antibiotic use are important to prevent quinolone resistance.

## Background

Quinolones, an important class of broad-spectrum antimicrobials against many Gram-negative aerobes, have been widely utilized since the first introduction of ciprofloxacin in 1987 [1]. Formerly, it was suggested that quinolones would be advantageous for minimizing resistance due to the rapid bactericidal activity. As the use of fluoroquinolones has increased, however, the occurrence of quinolone-resistant (QR) strains with decreased susceptibility has been increasingly reported. The QR microorganisms could be recovered from both nosocomial and community-acquired infections [2, 3].

The WHO has proclaimed antimicrobial resistance to be one of the greatest current threats to global health. It is generally accepted that antimicrobial resistance is directly associated with the use of antibiotics [4]. However, there are very few new antibiotic drugs in the pipeline [5], and current antibiotic drugs should be used prudently to decrease antimicrobial resistance rates [6].

*Escherichia coli* (*E. coli*) is the most common pathogen in both hospital and community settings, and the species can be divided into five major taxonomic lineages (A, B1, B2, D and E) [7]. The majority of *E. coli* strains are harmless, and they are even important for healthy people since they reside in the large intestine and provide their hosts with vitamin K complex [8]. Unfortunately, other pathogenic *E. coli* strains are capable of causing infections that can lead to serious consequences, including and urinary tract infection (UTI) [9] other extraintestinal infections [10]. Recent years, the quinolone-resistant *Escherichia coli* (QREC) has developed rapidly and spread widely [11]. Take Europe for example, according to the 2009 surveillance report of European Antimicrobial Resistance Surveillance Network (EARS-Net), 30% of all invasive *E. coli* isolates are resistant to FQ, which limits empirical treatment with these antimicrobials [12].

Knowledge of risk factors associated with QREC infection development is vital to identify high-risk patients in the prevention of QREC acquisition. In addition, such knowledge is also helpful for physicians to make a choice during the empirical therapeutic decision-making process and in the design of effective control measures to prevent infections. A lot of studies have been conducted to determine the risk factors for QREC infection, but the results are not always consistent. For example, Kratochwill et al. found that people with at least 65 years were significantly associated with QREC infection [13], while Pena et al. did not agree with that [14]. Therefore, we performed a systematic review and meta-analysis to investigate the risk factors for QREC infection based on the reported evidence.

## Methods

### Search strategy

A systematic literature search was conducted to collect the potential data in the electronic databases up to April 2019, including PubMed, EMBASE, and the Cochrane Library. Besides, the reference lists of retrieved reports and relevant systematic reviews were also collected and identified. Both subject terms and free terms were used in the search strategy, including terms related to risk factor assessment (risk/causes, risk factors/assessment, logistic models, multivariate analysis), drug-resistant (quinolone-resistant, fluoroquinolone-resistant), the pathogen (*Escherichia coli*), and mode of infection (nosocomial, hospital-acquired/associated, healthcare-acquired/associated). Unpublished data or grey literature searches were not performed. No study types were restricted during the search.

### Eligibility criteria

Studies were eligible when they met the following entry criteria: (1) about QREC, which was defined as the resistance of *Escherichia coli* to quinolone, fluoroquinolone, ciprofloxacin, norfloxacin, ofloxacin, fleroxacin, nalidixic acid or other quinolone agents; (2) about QREC infection; (3) studies were about the risk factors for QREC; (4) case-control studies, cohort studies and any other study designs except non-comparative studies, case reports, and case series; (5) studies with raw data of the risk factors; (6) patients with either hospital-acquired healthcare-acquired or community-acquired; (7) sample size > 20. No sex, age or ethnicity of patients were restricted. It should be noted that all the studies that evaluated the risk factors for QREC with control groups were eligible for this meta-analysis. No specific infection condition was restricted. No minimum study duration or follow-up time was required for inclusion. We excluded studies of mixed infections that had Gram-positive infections or any other Gram-negative infections beyond *Escherichia coli*. Studies published in languages other than English or Chinese were excluded. These eligibility criteria were verified based on the search results.

### Data extraction

Two review researchers independently performed the data extraction from included trials according to a previously created form. The extracted data included (1) the first author and years of publication; (2) study design; (3) years when patients were enrolled; (4) the country where the study was implemented; (4) number of cases and control patients; (5) number of male sex and age of cases and control patients; (6) drug resistance pattern and (7) all identified risk factors of QREC infection and other outcomes of interest. The extraction results were evaluated by another author and the disagreement between two researchers was resolved by discussion with another author.

### Quality assessment

The Newcastle-Ottawa Quality Assessment Form [15] was used to evaluate the quality of evidence for the included case-control and cohort studies. This standard form consists of three domains, i.e., selection (4 items, 4 points), comparability (1 item, 2 points), and outcome (3 items, 3 points). There are 8 items which scored a total of 9 points. A high-quality study scored 8–9 points, whereas a low-quality study scored less than 6 points. Other studies scored 6–7 points were rated as a moderate grade. Two authors evaluated the quality of each study included in this meta-analysis. Uncertainty or disagreement was resolved by discussion to reach a consensus.

### Outcome measures

The present meta-analysis focused on the risk factors for QREC infection. We collected all risk factors when at least two studies reported it. The demographic factors included age, sex (male) and race. The comorbidities included cancer, hepatic cirrhosis, diabetes mellitus, HIV/AIDS (human immunodeficiency virus infection and acquired immune deficiency syndrome), cerebrovascular disease, cardiovascular disease, neurogenic bladder, renal dysfunction, transplantation, urinary tract abnormality, dementia, COPD (chronic obstructive pulmonary diseases), congestive heart failure, autoimmune disease, connective tissue disease, gastrointestinal disease, neurologic disease and hypertension, respiratory disease. Both present treatments and prior treatment included urinary catheter and surgery. Besides, present treatments also included immunosuppressive drugs and nursing home, while prior treatment also included prior quinolones, prior other antibiotics, prior UTI (urinary tract infection) and prior hospitalization. Acquisition of infection source was also analyzed in this meta-analysis. Other parameters of prevalence and prognosis were narratively reviewed.

### Statistical analysis

Chi-squared test and the I^2^ statistics were adopted to evaluate the heterogeneity among the included studies [16, 17]. I^2^ ≥ 50% or *p* ≤ 0.1 was considered as a significant heterogeneity and a random-effects model was utilized. Otherwise, a fixed-effects model was used when I^2^ < 50%. Pooled odds ratios (ORs) and corresponding 95% CIs were calculated for the measurement of the risk factors of QREC infection. The Z-test was utilized to determine the significance of the pooled ORs. Reported probability values were two-sided, with significance set at *p* ≤ 0.05. Besides, sensitivity analyses were conducted through sequential omission of individual studies in each comparison. If the corresponding *p*-value of pooled ORs was not substantially different, the results of sensitivity were identified as credible. Potential publication bias was estimated by funnel plots. Symmetrical funnel plots were identified as credible. The present meta-analysis was performed using a Review Manager software, version 5.3 for Windows (Cochrane Collaboration, Oxford, United Kingdom).

## Results

### Study selection

**Fig. 1:**
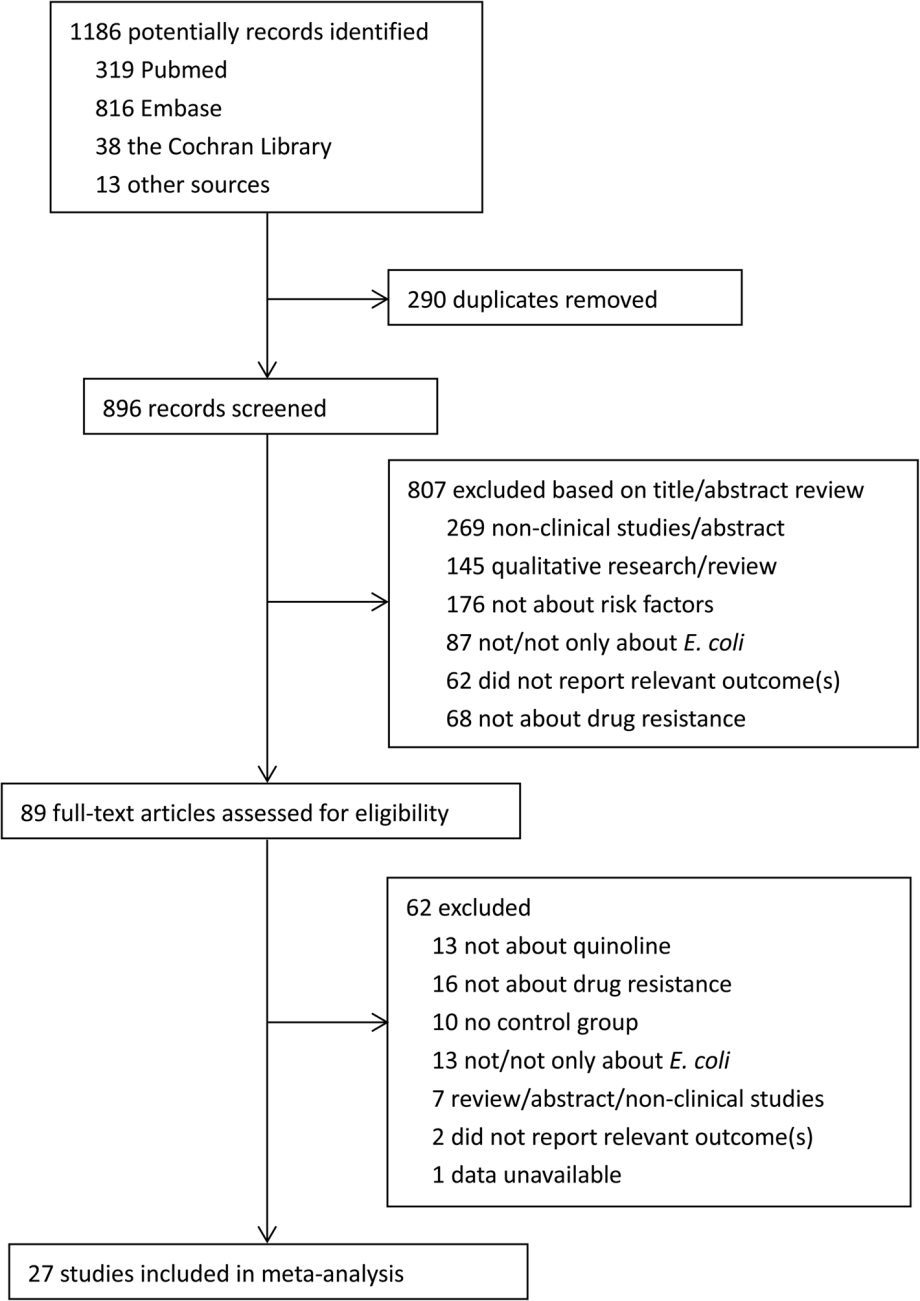
PRISMA flow chart of study selection in the meta-analysis

The literature selection process was performed as shown in the flow diagram of Preferred Reporting Items for Systematic Reviews and Meta-analyses (PRISMA) (Fig. 1). The literature search identified a total of 1186 citations from various electronic databases and the reference lists of retrieved studies and relevant systematic reviews. After removing duplicates using EndNote X7 software, 896 were unique records that were eligible for screening. Then, those potential studies were screened by two authors based on the title/abstract, and 89 were further identified for full-text screening. The disagreements were discussed with a third author to reach a consensus. Finally, 27 studies were included in the present meta-analysis [2, 3, 13, 14, 18–40].

### Study characteristics

The characteristics of the included studies were shown in Table 1. Of the studies included for qualitative synthesis, all were case-control studies [2, 3, 14, 18–27, 31, 32, 36, 40] and cohort studies [13, 28–30, 33–35, 37–39] in design published between 1995 and 2017. Three were conducted in Spain [2, 14, 32], five in South Korea [18, 20, 30, 36, 37], two in France [19, 35], nine in the USA [13, 21–23, 25, 27, 33, 34, 39], two in the Netherlands [31, 40], one each in Finland [3], Israel [24], China [26], the UK [28], Turkey [29], and Pakistan [38]. A total of 67,019 participants (2526 cases and 64,493 controls) were enrolled in this meta-analysis. The sample sizes in included studies ranged from 49 [23] to 59,469 [39]. Most studies focused on the *E. coli* resistance patterns for ciprofloxacin or levofloxacin-resistant strains. Outcomes of interest included the demographic data and all risk factors, including comorbidities or underlying diseases among the study populations, present and prior treatments, infection source and so on. Besides risk factors of QREC infection, some studies also reported the prevalence [3, 29, 30, 32, 33] and prognosis due to QREC [18].

**Table 1:** Characteristics of the eligible studies

Study (year)	Study design	Years enrolled	Study region	Case/control, n	Male sex, case/control, n	Mean age (SD or range), case/control, years	Drug resistance pattern	Risk of bias^a^	Outcomes of interest
Pena et al. (1995)	Case- control	1988–1992	Spain	27/54	15/24	N/A	Ciprofloxacin-resistant	3/1/2/6	Age, underlying diseases, acquisition, source of infection, immunosuppressive drugs, prior quinolones, prior other antibiotics
Garau et al. (1999)	Case- control	1992–1997	Spain	70/502	44/264	70.2(13)/ 65.8(18.4)	Ciprofloxacin-resistant	4/2/2/8	Age, sex, underlying disease, acquisition, source of infection, urinary catheter, prior antibiotic use, prior quinolone use, mortality
Cheong et al. (2001)	Case- control	1993–1998	Korea	40/80	28/33	51/54	Norfloxacin or ciprofloxacin-resistant	4/1/2/7	Age, sex, chronic underlying disease, source of bacteremia, urinary catheter, prior antibiotic use acquisition, APACHE II score, duration of antibiotic treatment, death
Sotto et al. (2001)	Case- control	1998–1999	France	17/303	7/67	N/A	Norfloxacin, pefloxacin, ofloxacin, or ciprofloxacin-resistant	4/1/2/7	Age, sex, unit of hospitalization, urinary catheter, nosocomial acquisition, prior hospitalization, prior UTI, prior urinary catheter, prior antibiotic exposure
Eom et al. (2001)	Case- control	1996–2000	Korea	60/80	16/24	58/40	Ciprofloxacin-resistant	4/1/2/7	Age, sex, UTIs, chronic underlying illness, urinary catheter, neurogenic bladder, recurrent UTI, previous admission, operation, prior use of fluoroquinolone, prior use of other antibiotics, duration of previous antibiotics, site of acquisition, hospitals, mortality
Lautenbach et al. (2002)	Case- control	1998–1999	USA	123/70	52/20	75 (32–100)/ 67 (22–99)	Levofloxacin-resistant	4/2/2/8	Age, hospital, admitted from long-term care facility, race, admitted from outside hospital diarrhea, sex, APACHE II score, hospitalized in past 30 d, hospital duration, central venous catheter, urinary catheter, mechanical ventilation, patient location
Huotari et al. (2003)	Case- control	1997–1999	Finland	51/102	21/31	62.6 (20–90)/ 67.1 (21–96)	Norfloxacin or ciprofloxacin-resistant	4/1/1/6	Age, time from admission to isolation in days, sex, prior fluoroquinolone therapy, prior therapy with other antimicrobial agent, urinary tract abnormalities, immunosuppression, surgery, organ transplant
Killgore et al. (2004)	Case- control	2001.01–12	USA	40/80	7/11	61 (59)/ 51 (53)	Ciprofloxacin-resistant	4/1/2/7	Age, sex, urinary tract symptoms, previous ciprofloxacin use, previous use of quinolone, previous use of other antibiotic, urinary tract abnormality, catheter, comorbidity, previous surgery, previous hospitalization, recurrent UTI
Maslow et al. (2005)	Case- control	2002.02–07	USA	25/24	24/23	73 (38–87)/ 65.5 (42–98)	Fluoroquinolone-resistant	4/1/1/6	prior hospitalization, duration of residence in facility, decubitus ulcer, low ambulatory status, fluoroquinolone use, prior metronidazole use
Colodner et al. (2008)	Case- control	2005.07–10	Israel	150/150	16/37	57 (18–92)/ 71 (19–94)	Ciprofloxacin or ofloxacin-resistant	4/1/1/6	Age, sex, prior hospitalization, clinical status (dementia, other neurological disease, diabetes mellitus type 2, cardiovascular disease, etc)
Johnson et al. (2008)	Case- control	1998–2005	USA	41/82	N/A	55.9/55.4	Levofloxacin-resistant	4/2/2/8	Diabetes, congestive heart failure, any catheter use, any levofloxacin use, or any surgical procedure
Lin et al. (2008)	Case- control	1999.09–12	China	61/122	24/35	62.4 (22.1)/ 48.2 (28.2)	Ciprofloxacin-resistant	3/1/2/6	Demographic characteristics, underlying disease, medical devices, antibiotics administration
Lautenbach et al. (2009)	Case- control	2002–2004	USA	89/685	54/363	66 (54–74)/ 61 (49–73) ^#^	Levofloxacin-resistant	4/1/1/6	Age, sex, race, prior hospitalization, comorbidities, prior antimicrobial use
Rooney et al. (2009)	Cohort studyd	2004–2006	UK	119/175	28/44	N/A	Ciprofloxacin-resistant	3/1/2/6	MRSA or infection, antibiotic use, rimethoprim use, fluoroquinolone use, UTI, hospitalization, catheter use
Yagci et al. (2009)	Cohort study	N/A	Turkey	32/104	12/41	51(13)/ 57(16)	Ciprofloxacin, moxifloxacin, or levofloxacin-resistant	2/1/2/5	Age, sex, race, comorbidities, fluoroquinolone use, hospitalization
Jang et al. (2011)	Cohort study	2005–2009	Korea	509/192	N/A	N/A	Levofloxacin-resistant	2/1/2/5	medical history, underlying disease, status of urinary catheterization
van et al. (2011)	Case- control	2004–2009	The Netherlands	51/369	18/119	71 (54–80)/ 66 (44–78) ^#^	Ciprofloxacin-resistant	4/1/2/7	Age, sex, comorbidities, UTI, hospitalisation, residence in nursing home, antimicrobial use, patient environment characteristics
Smithson et al. (2012)	Case- control	2008–2011	Spain	52/101	N/A	66(16.6)/ 58(16.9)	Ciprofloxacin or levofloxacin-resistant	4/1/2/7	Age, HA-UTI, comorbidities, previous antibiotic treatment
Bailey et al. (2013)	Cohort study	2009–2011	USA	39/183	13/28	N/A	Levofloxacin-resistant	2/1/2/5	Sex, race, comorbidities, home use of antibiotics, surgical procedures
Han et al. (2013)	Cohort study	2002–2004	USA	73/322	36/180	63.2(17.6)/ 61.3(15.1)	Levofloxacin-resistant	3/1/2/6	Age, sex, race, surgical procedures, residence in nursing home, comorbidities
Bedoin et al. (2014)	Cohort study	2011–2012	France	60/284	0/0	76.6/70.2	Ofloxacin-resistant	2/2/3/7	Demographic data, administrative data, clinical data, therapeutic data
Kim et al. (2014)	Case- control	2000–2011	Korea	26/56	19/40	58.2 (9.6)/ 57.8 (10.4)	Ciprofloxacin-resistant	4/1/2/7	Age, sex, cause of cirrhosis, Child-Pugh classification, comorbidities, use of antibiotics, hospitalization, mortality
Park et al. (2014)	Cohort study	2012.04–06	Korea	67/162	0/0	71 (59–77)/ 67 (50–76) ^#^	Ciprofloxacin- resistant	3/2/2/7	Age, comorbidities, bed-ridden state, use of antibiotics, APN, UTI, isolation of CIP-resistant *E. coli* in the urine
Jadoon et al. (2015)	Cohort study	2011–2012	Pakistan	66/100	N/A	N/A	Ciprofloxacin- resistant	3/1/3/7	Recurrent UTI, history of prior use of ciprofloxacin, diabetes mellitus, immuno-suppressive agent use, history of catheterization
Kratochwill et al. (2015)	Cohort study	2011–2014	USA	100/100	19/10	52.6 (21.7)/ 38.0 (18.4)	Ciprofloxacin-resistant	4//1/2/7	Previous antibiotic use, residence in nursing home, chronic indwelling catheter, recent hospitalization, recurrent UTIs, male sex, age
Saade et al. (2016)	Cohort study	2000–2013	USA	428/59041	N/A	64.9 (25–97)/ 65.0 (44–93)	Ciprofloxacin or levofloxacin-resistant	4/2/2/8	Age, diabetes, history of a culture positive for FQ-resistant E. coli, admission, fluoroquinolone use, other antibiotic use
Mulder et al. (2017)	Case- control	2000–2006	The Netherlands	110/970	28/185	79 (52)/ 73 (65)	Ciprofloxacin-resistant	4/1/2/7	Age, sex, BMI, kidney function, diabetes, SES, fluoroquinolone use, timing of last fluoroquinolone prescription, duration of last prescription

^a^ Cohort and case-control studies were assessed by the Newcastle-Ottawa Quality Assessment Scale. The scores are presented as selection/comparability/outcome/total score

^**#**^ Age, median (IQR), years

Abbreviations: *N/A* not applicable, *AC* acute-care, *IC* intermediate-term-care, *LC* long-term-care (> 1 month), *UTI* urinary tract infections, *COPD* chronic obstructive pulmonary disease, *APACHE* Acute Physiology, and Chronic Health Evaluation, *MRSA* ethicillin-resistant *Staphylococcus aureus*, *HA-UTI* healthcare-associated urinary tract infection, *APN* acute pyelonephritis, *BMI* body mass index, *SES* socioeconomic status

### Study quality

For the 27 observational studies evaluated by Newcastle-Ottawa Quality Assessment Form, four studies scored 8 points [2, 21, 25, 39], which could be regarded as at high-quality. While three study only scored 5 points [29, 30, 33], and could be regarded as at low-quality. The remaining 20 studies scored 6–7 points and could be regarded as at moderate-quality. Therefore, most studies were of moderate-to-high quality. They generally lost points because of a statement of the outcome of interest at the beginning and non-complete follow up.

### Risk factors for QREC infection

**Fig. 2:**
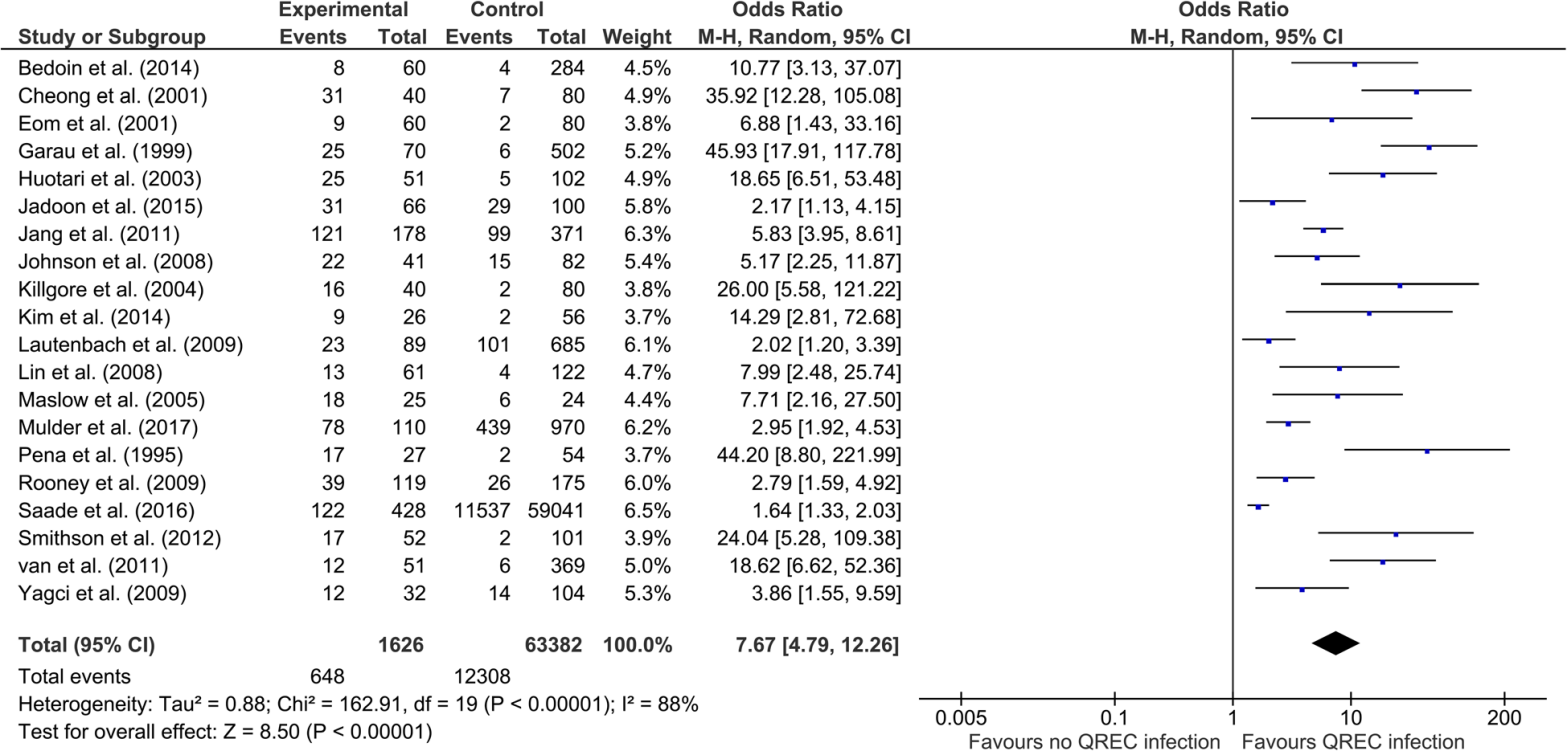
Forest plots for the association between exposure to quinolone and QREC infection. QREC, quinolone-resistant *Escherichia coli*; M-H, Mantel-Haenszel; CI, confidence interval

Table 2 shows the pooled results of risk factors for QREC infection and heterogeneity in the meta-analysis. For the demographic parameters, the results showed that gender (male) [OR (95% CI) = 1.41 (1.21 to 1.64), *p* <  0.001] is the primary risk factor. However, our evidence did not support that the remaining demographic factors were risk factors for QREC infection. For the comorbidities, the pooled results showed demonstrated that some comorbidities might increase the risk for QREC infection, including hepatic cirrhosis [OR (95% CI) = 2.05 (0.99 to 4.24), *p* <  0.05], diabetes mellitus [OR (95% CI) = 1.62 (1.43 to 1.83), *p* <  0.001], cardiovascular disease [OR (95% CI) = 1.76 (1.02 to 3.04), *p* = 0.04], neurogenic bladder [OR (95% CI) = 8.66 (5.68 to 13.19), *p* <  0.001], renal dysfunction [OR (95% CI) = 2.47 (1.44 to 4.23), *p* = 0.001], transplantation [OR (95% CI) = 2.37 (1.17 to 4.79), *p* = 0.02], urinary tract abnormality [OR (95% CI) = 1.85 (1.37 to 2.49), *p* <  0.001], dementia [OR (95% CI) = 5.83 (2.33 to 14.60), *p* <  0.001], congestive heart failure [OR (95% CI) = 5.63 (1.27 to 25.10), *p* = 0.02], neurologic disease [OR (95% CI) = 2.80 (1.71 to 4.57), *p* <  0.001]. For the treatments, the significant risk factors included immunosuppressive drugs [OR (95% CI) = 2.02 (1.43 to 2.85), *p* <  0.001], urinary catheter [OR (95% CI) = 4.39 (2.81 to 6.85), *p* <  0.001] and nursing home resident [OR (95% CI) = 4.63 (1.62 to 13.26), *p* = 0.004]. Besides, a lot of prior treatments could also be regarded as the risk factors, including prior surgery [OR (95% CI) = 2.54 (1.28 to 5.04), *p* = 0.008], quinolones [OR (95% CI) = 7.67 (4.79 to 12.26), *p* <  0.001], other antibiotics [OR (95% CI) = 2.74 (1.92 to 3.92), *p* <  0.001], UTI [OR (95% CI) = 2.79 (2.32 to 3.36), *p* <  0.001] and hospitalization [OR (95% CI) = 2.06 (1.62 to 2.60), *p* <  0.001]. Furthermore, nosocomial acquisition of infection was also a significant risk factor for QREC [OR (95% CI) = 2.35 (1.47 to 3.75), *p* <  0.001]. No significant difference of infection source was found between case group and control group (all *p* > 0.05), suggesting that various infection sources were not the risk factors. Figure 2 illustrates the forest plot describing the relationship between quinolones exposure and QREC infection.

**Table 2:** Pooled risk factors for QREC infections

Risk factors	No. of studies	No. of case/control	Meta-analysis		Model	Test of heterogeneity	
			OR (95% CI)	*p* value		I^2^	p value
Demographic factors							
Age (> 65 years)	3	144/457	2.42 (0.45, 12.97)	0.30	R	86%	< 0.001
Gender (male)	18	1155/4437	**1.41 (1.21, 1.64)**	**< 0.001**^a^	F	33%	0.09
Race (white)	4	301/1290	1.19 (0.57, 2.48)	0.65	R	85%	< 0.001
Comorbidities							
Cancer	12	722/1886	1.66 (0.96, 2.85)	0.07	R	75%	< 0.001
Hepatic cirrhosis	3	127/214	**2.05 (0.99, 4.24)**	**0.05**	F	7%	0.34
Diabetes mellitus	18	1598/62968	**1.62 (1.43, 1.83)**	**< 0.001**	F	24%	0.17
HIV/AIDS	3	189/1061	2.89 (0.54, 15.34)	0.21	R	54%	0.11
Cerebrovascular accident	4	329/860	1.60 (0.75, 3.44)	0.22	R	68%	0.02
Cardiovascular disease	4	351/756	**1.76 (1.02, 3.04)**	**0.04**	R	69%	0.02
Neurogenic bladder	2	238/411	**8.66 (5.68, 13.19)**	**< 0.001**	F	0	0.55
Renal dyfunction	11	768/3117	**2.47 (1.44, 4.23)**	**0.001**	F	78%	< 0.001
Transplantation	3	164/504	**2.37 (1.17, 4.79)**	**0.02**	F	19%	0.29
Urinary tract abnormality	3	269/780	**1.85 (1.37, 2.49)**	**< 0.001**	F	0	0.70
Dementia	2	202/251	**5.83 (2.33, 14.60)**	**< 0.001**	F	0	0.89
COPD	6	332/916	1.30 (0.81, 2.09)	0.27	F	0	0.55
Congestive heart failure	2	93/183	**5.63 (1.27, 25.10)**	**0.02**	F	0	0.97
Autoimmune disease	3	242/601	1.06 (0.20, 5.50)	0.95	R	54%	0.12
Connective tissue disease	2	128/284	0.47 (0.10, 2.19)	0.34	F	0	0.56
Gastrointestinal disease	2	239/456	1.19 (0.61, 2.34)	0.61	R	57%	0.13
Neurologic disease	2	128/284	**2.80 (1.71, 4.57)**	**< 0.001**	F	0	0.77
Hypertension	2	217/514	2.70 (0.94, 7.76)	0.07	R	84%	0.01
Respiratory disease	3	172/789	2.08 (0.56, 7.74)	0.27	R	82%	0.004
Present treatments							
Immunosuppressive drugs	9	499/1328	**2.02 (1.43, 2.85)**	**< 0.001**	F	18%	0.28
Urinary catheter	15	1119/2958	**4.39 (2.81, 6.85)**	**< 0.001**	R	68%	< 0.001
Surgery	4	303/456	1.76 (0.42, 7.43)	0.44	R	90%	< 0.001
Nursing home resident	5	315/1075	**4.63 (1.62, 13.26)**	**0.004**	R	61%	0.03
Prior treatments							
Prior surgery	6	417/1050	**2.54 (1.28, 5.04)**	**0.008**	R	68%	0.008
Prior quinolones	20	1626/63382	**7.67 (4.79, 12.26)**	**< 0.001**	R	88%	< 0.001
Prior other antibiotics	21	1726/63862	**2.74 (1.92, 3.92)**	**< 0.001**	R	84%	< 0.001
Prior urinary catheter	2	81/355	2.01 (0.35,11.48)	0.43	R	74%	0.05
Prior UTI	14	939/2402	**2.79 (2.32, 3.36)**	**< 0.001**	R	73%	< 0.001
Prior hospitalization	16	1448/62158	**2.06 (1.62, 2.60)**	**< 0.001**	R	53%	0.009
Acquisition							
Nosocomial	7	307/1245	**2.35 (1.47, 3.75)**	**< 0.001**	R	56%	0.03
Infection sources							
Urinary tract	3	137/636	0.78 (0.28, 2.19)	0.64	R	81%	0.006
Intra-abdominal	2	110/582	0.88 (0.53, 1.46)	0.62	F	0	0.95
Respiratory tract infection	2	80/160	1.55 (0.51, 4.66)	0.44	F	0	0.74

^a^ Statistically significant values (*p* ≤ 0.05)

Abbreviations: *QREC* quinolone-resistant *Escherichia coli*, *OR* odds ratio, *R* random effect model, *F* fixed effect model, *HIV/AIDS* human immunodeficiency virus infection and acquired immune deficiency syndrome, *COPD* chronic obstructive pulmonary diseases, *UTI* urinary tract infection

### Sensitivity analyses

Sensitivity analyses were used to find out the obvious study which could change the pooled results through the result of itself. Thus, we conducted sensitivity analyses among the comparisons of the results of pooled ORs for the random-effects and fixed-effects models by the sequential and one-by-one omission of individual studies. Our sensitivity analyses revealed that the corresponding results were not significantly different in most comparisons of the risk factors, suggesting that our meta-analyses were stable. Besides, we even found that the heterogeneity was reduced in some conditions during the sensitivity analyses. However, some studies did change the pooled results. Specifically, when we removed the studies of Han et al., [34] the heterogeneities for cancer and cardiovascular disease were distinctly reduced evidently by I^2^ decreased from 75 to 40% and from 69 to 46%, respectively. After using a fixed-effects model, the ORs and the corresponding 95% CIs for cancer changed from 1.66 (0.96, 2.85) to 2.07 (1.57, 2.75) and from 1.76 (1.02, 3.04) to 2.28 (1.66, 3.12). respectively. Similarly, when we removed the study of Pena et al., [14] the ORs (95% CIs) for hepatic cirrhosis and urinary tract infection changed from 2.05 (0.99, 4.24) to 2.96 (1.22, 7.15) and from 0.78 (0.28, 2.19] to 0.48 (0.31, 0.74), respectively. These corresponding results changed and became statistically significant.

### Publication bias

**Fig. 3:**
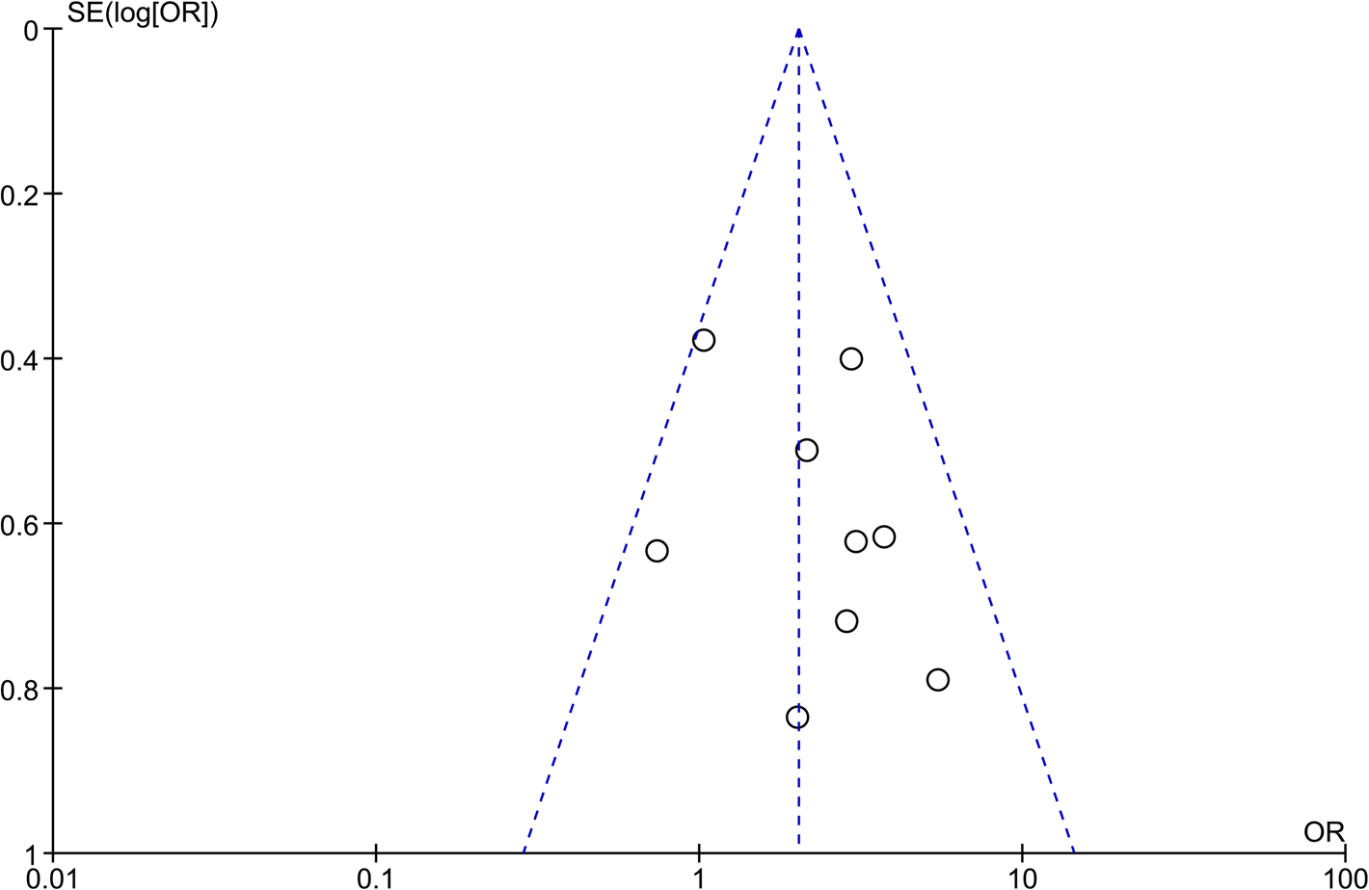
Funnel plot of comparison for immunosuppressive drugs uses as the potential risk factor. Each small circle represents an independent study for the indicated association. The funnel plot appears asymmetric. OR, odds ratio; SE, standard error

The publication bias among the included studies was evaluated using funnel plots. No obvious asymmetry was identified in funnel plots of most outcomes, suggesting that there was no significant publication bias. However, the publication bias of immunosuppressive drugs use should not be ignored. As shown in Fig. 3, the funnel plot was quite asymmetrical.

## Discussion

In the current systematic review and meta-analysis, we aimed to evaluate the risk factors for QREC infection by summarizing the published data of relevant articles so far. Since it was first evaluated in Japan in 1988 [41], QREC has become one of the most frequent QR bacterial strains, which has also been endemic in other areas worldwide, including East Asia [26], South Asia [38], Europe [19, 35], and North America, especially the USA [21–23, 25, 27, 33, 34, 39]. The direct consequence of QREC dissemination is that infections with such strains are difficult to eradicate and the treatment options are limited. Thus, from the perspective of preventive medicine, determining the possibility of QREC infection in the early stage by studying risk factors and taking reasonable prevention could be helpful to reduce the incidence. Although some studies [2, 14, 18] on the risk factors of QREC infection are previously available, their results are controversial because of the different selection criteria, sample sizes or study designs. Accordingly, we performed the present meta-analysis, which to our knowledge, is the first meta-analysis in this field, to identify the potential risk factors for QREC infection.

After a comprehensive search, 27 case-control or cohort studies with 67,019 participants with infections were collected in our meta-analysis. Based on the outcomes reported in these included studies, we pooled the data of each risk factor according to the classification. Our meta-analysis identified that the risk factors for acquisition of QREC infection varied about demographic factors, comorbidities, present and prior treatments as well as acquisition routes. Infection sources, including urinary tract, intra-abdominal and respiratory tract infection were not identified as the significant risk factors.

Specifically, our meta-analysis did not suggest the old people (> 65 years) as the risk factor. But heterogeneity could not be ignored. The sensitivity analysis demonstrated that when the study of Pena et al. [14] was omitted, the old people (> 65 years) were more susceptible to QREC infection. Besides, since some original studies compared the age using continuous data with mean ± standard deviation, we conducted another comparison using the pooled mean difference (MD) with 95% CI. As was shown in Additional file 1: Figure S1, the meta-analysis found that age was a significant risk factor for QREC infection [MD (95% CI) = 5.21 (0.39 to 10.03), *p* = 0.03].

Over twenty comorbidities or underlying diseases were reported in the original studies. Our findings suggest that some comorbidities were significantly associated with the acquisition of QREC infection compared with *E. coli*. These noteworthy comorbidities included hepatic cirrhosis, diabetes mellitus, cardiovascular disease, neurogenic bladder, renal dysfunction, transplantation, urinary tract abnormality, dementia, congestive heart failure, neurologic disease.

Some treatments may also closely relate to the production of QREC infection. Among them, immunosuppressive drugs use, urinary catheter use, hospitalization stay and prior quinolones, other antibiotics use were identified as significant risk factors. Therefore, no matter exposure to quinolones or other antibiotics, the risk of acquisition QREC infection is increased. On the other hand, it is also worth mentioning that QREC isolates may also be resistant to other antimicrobial agents. For example, Lin and the colleagues found that 98.4% ciprofloxacin-resistant *E. coli* strains were concurrently resistant to at least one of eight antimicrobial classes other than ciprofloxacin, including monobactams, carbapenems, penicillins, cephalosporins, aminoglycosides, minocycline, TMP/SMX, and chloramphenicol [26]. Therefore, It is necessary to use antibiotics carefully and appropriately. Clinicians in different countries should be aware of local antimicrobial resistance data and prescribe or adjust antibiotics according to susceptibility testing results.

In addition, some original studies also compared the outcomes between cases and controls [18, 20]. Generally, the prognosis is poorer among case patients compared with control patients. Take mortality for example, patients infected with QREC have a higher overall rate of mortality than the control patients (25.51 vs 12.67%). Pooling data from four original studies according to the mortality gave odds ratio of 2.43 (1.59 to 3.72) with *p* <  0.001 for patients infected with QREC vs. control patients (Additional file 2: Figure S2).

There are several strengths in this systematic review and meta-analysis. First, the full text of meta-analysis was designed and reported closely followed the standard PRISMA guidelines, which were comprehensible and concise for other peer researchers in this field. And to our knowledge, this study is the first comprehensive meta-analysis to date focusing on the risk factors for QREC infections. Second, considering there are numerous potential risk factors, we summarized each of them once two or more studies contained. The comprehensive reports of comparisons contribute to finding the shortages in these included studies. Last but not least, 27 studies with 67,019 participants were included in this meta-analysis. The huge sample sizes are conducive to guarantee statistical power and reliability of the results of this meta-analysis.

As with many systematic reviews and meta-analyses, our study has some limitations, which may affect the results. First, as most of the included studies only reported unadjusted data on risk factors, we analyzed only crude risk factors among participants with QREC in the section of results. Second, the present study only retrieved published studies from three database and the relevant studies from the reference lists. Other potential articles published in other databases and unpublished studies might have been missed. Third, the significant heterogeneity (I^2^ ≥ 50% or *p* ≤ 0.1) was identified in some variables across these eligible original trials. The heterogeneity may be derived from the different study design (cohort studies and case-control studies) or antimicrobial susceptibility testing cannot be ignored in some comparisons.

## Conclusions

In conclusion, the present meta-analysis has identified a number of factors associated with QREC infection development. Therefore, these results have some implication for the medical institutions and the centers for disease control that attention should be paid to patients with QREC infection. Further studies are required in order to better stratify and control quinolone resistance risk in patients with *E. coli* infections.

## Supplementary information

**Additional file 1: Figure S1.** Forest plots of pooled age as the potential risk factor for QREC infection. QREC, quinolone-resistant *Escherichia coli*; I-V, Inverse-Variance; CI, confidence interval.

**Additional file 2: Figure S2.** Forest plots of pooled mortality in participants infected with *Escherichia coli*. I-V, Inverse-Variance; CI, confidence interval.

## Data Availability

Data supporting the conclusions of this article are available from the corresponding author on reasonable request.

## Abbreviations

CI: Confidence Interval
COPD: Chronic Obstructive Pulmonary Diseases
*E. coli*: *Escherichia coli*
EARS-Net: European Antimicrobial Resistance Surveillance Network
HIV/AIDS: Human Immunodeficiency Virus infection and Acquired Immune Deficiency Syndrome
MD: Mean Difference
OR: Odds Ratios
PRISMA: Preferred Reporting Items for Systematic Reviews and Meta-analyses
QR: Quinolone Resistant
QREC: Quinolone-Resistant *Escherichia coli*
TMP/ SMX: Trimethoprim/Sulfamethoxazole
UTI: Urinary Tract Infection
