# Supplementary figures and images for: Risk factors for quinolone-resistant Escherichia coli infection: a systematic review and meta-analysis
Source: Antimicrob Resist Infect Control. 2020 Jan 9;9:11. doi: 10.1186/s13756-019-0675-3 (PMC6953284; doi:10.1186/s13756-019-0675-3)

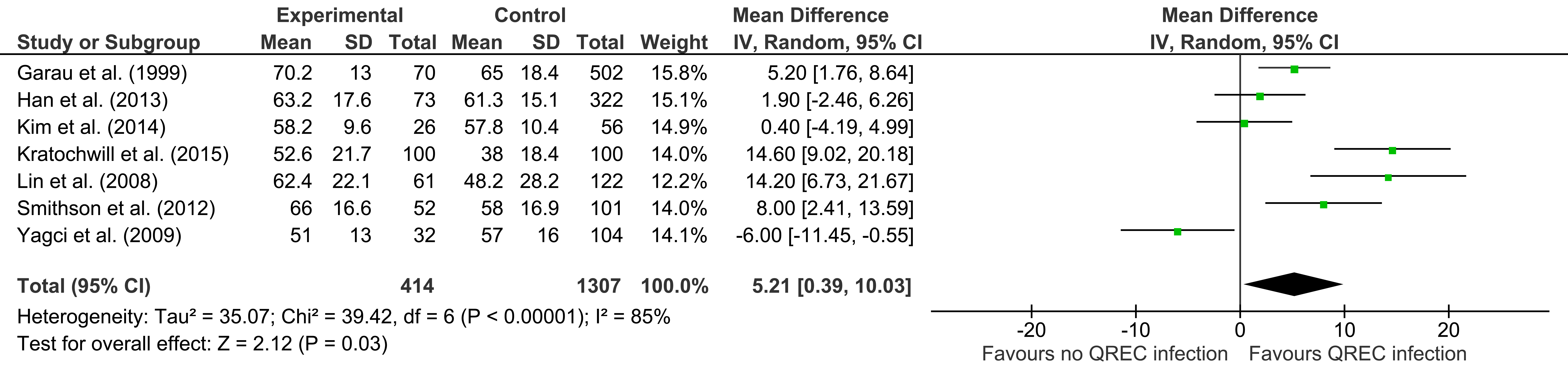

Supplement: Supplementary file 1 — Additional file 1: Figure S1. Forest plots of pooled age as the potential risk factor for QREC infection. QREC, quinolone-resistant Escherichia coli; I-V, Inverse-Variance; CI, confidence interval. [file 13756_2019_675_MOESM1_ESM.tif]

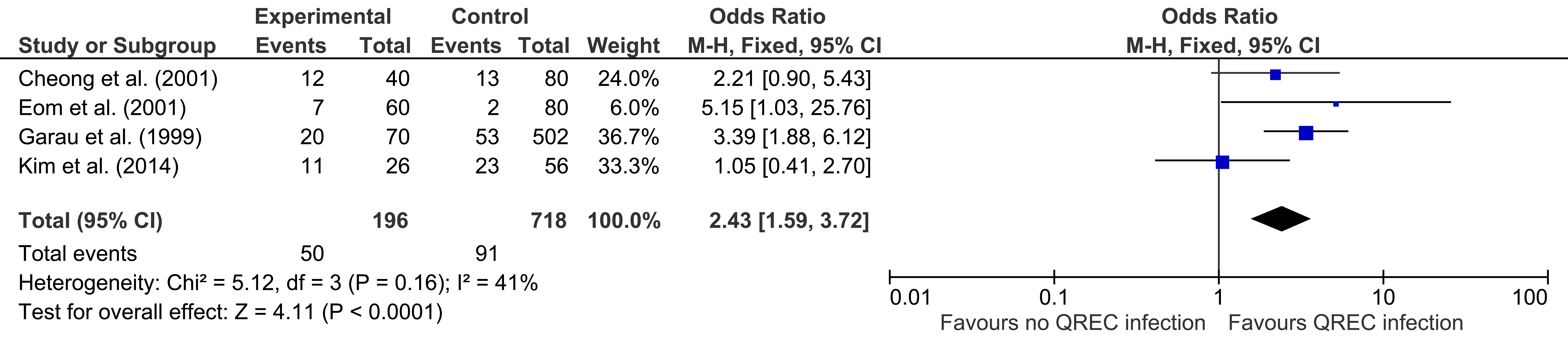

Supplement: Supplementary file 2 — Additional file 2: Figure S2. Forest plots of pooled mortality in participants infected with Escherichia coli. I-V, Inverse-Variance; CI, confidence interval. [file 13756_2019_675_MOESM2_ESM.tif]
